# Beyond the single index: Investigating ecological mechanisms underpinning ecosystem multifunctionality with network analysis

**DOI:** 10.1002/ece3.7987

**Published:** 2021-08-24

**Authors:** Ewa Siwicka, Rebecca Gladstone‐Gallagher, Judi E. Hewitt, Simon F. Thrush

**Affiliations:** ^1^ Institute of Marine Science University of Auckland Auckland New Zealand; ^2^ Tvärminne Zoological Station University of Helsinki Hanko Finland; ^3^ National Institute of Water and Atmospheric Research (NIWA) Hamilton New Zealand; ^4^ Department of Statistics University of Auckland Auckland New Zealand

**Keywords:** coastal ecology, ecosystem complexity, ecosystem multifunctionality, network analysis, species richness

## Abstract

Ecosystems simultaneously deliver multiple functions that relate to both the activities of resident species and environmental conditions. One of the biggest challenges in multifunctionality assessment is balancing analytical simplicity with ecosystem complexity. As an alternative to index‐based approaches, we introduce a multivariate network analysis that uses network theory to assess multifunctionality in terms of the relationships between species' functional traits, environmental characteristics, and functions. We tested our approach in a complex and heterogeneous ecosystem, marine intertidal sandflats. We considered eight ecosystem function, five macrofaunal functional trait groups derived from 36 species, and four environmental characteristics. The indicators of ecosystem functions included the standing stock of primary producers, oxygen production, benthic oxygen consumption, DIN (ammonium and NOx efflux) and phosphate release from the sediments, denitrification, and organic matter degradation at the sediment surface. Trait clusters included functional groups of species that shared combinations of biological traits that affect ecosystem function: small mobile top 2 cm dwellers, suspension feeders, deep‐dwelling worms, hard‐bodied surface dwellers, and tube‐forming worms. Environmental characteristics included sediment organic matter, %mud, %shell hash, and %sediment water content. Our results visualize and quantify how multiple ecosystem elements are connected and contribute to the provision of functions. Small mobile top 2 cm dwellers (among trait clusters) and %mud (among environmental characteristics) were the best predictor for multiple functions. Detailed knowledge of multifunctionality relationships can significantly increase our understanding of the real‐world complexity of natural ecosystems. Multivariate network analysis, as a standalone method or applied alongside already existing single index multifunctionality methods, provides means to advance our understanding of how environmental change and biodiversity loss can influence ecosystem performance across multiple dimensions of functionality. Embedding such a detailed yet holistic multifunctionality assessment in environmental decision‐making will support the assessment of multiple ecosystem services and social‐ecological values.

## Graphical abstract

Our manuscript presents the method, multivariate network analysis, that allows studying complex mechanistic ecological underpinnings of ecosystem multifunctionality at the ecosystem scale, overcoming information loss generated by commonly used indices such as species richness.

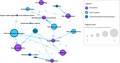

## 1 INTRODUCTION

Advancing our understanding of complex functional mechanisms underpinning natural ecosystems is needed to inform a more holistic approach to environmental management (Felipe‐Lucia et al., 2020). However, simultaneously assessing multiple ecosystem functions is a nontrivial task. Conceptually, a multifunctional assessment should truly represent the complexity of natural ecosystems. In practical terms, many real‐world ecosystems are dynamic, heterogeneous and contain a diverse array of species, which represent a major challenge to multifunctional assessments (Cardinale et al., 2012; Lefcheck et al., 2015). Historically, functions were assessed in isolation (Byrnes et al., 2014; Hector & Bagchi, 2007; Reiss et al., 2009). Only recently has the concept of multifunctionality, defined as a simultaneous performance of many functions, been advanced in ecological research (Manning et al., 2018). Nevertheless, the available methods that enable assessments of multiple functions (e.g., univariate assessment of individual functions or multifunction indices) deliver a limited amount of detail on the ecology that underpins the provisioning of functions (see method reviews by Byrnes et al., 2014, and Dooley et al., 2015). This loss of information on functions is also compounded by the use of species richness, as a descriptor of community structure or biodiversity. Species richness has a long history of use in ecology and has certainly retained its usefulness, and however, its application does need to be tempered by context. In the context of multifunctionality assessments, aggregation of functionally different species as a univariate measure reduces our ability to truly capture the role of specific species or groups of species that share specific functional traits. Species richness treats all species equally and variation in their abundance and unequal roles in functional regulation are ignored (Bradford et al., 2014). This limits ecological insight and the sensitivity of these approaches to assess the consequences of change (Bradford et al., 2014).

A more accurate assessment of how ecological communities contribute to functions can be facilitated by looking directly at species traits, that is, the species phenotype or behavior that influences ecosystem processes (Cadotte et al., 2011; Duncan et al., 2015; Hooper et al., 2005). Functional traits allow us to look more directly at the relationships between species and functions and, therefore, have been demonstrated to be a better proxy for functional (Cadotte et al., 2011; Hewitt et al., 2008; Petchey et al., 2004). Nevertheless, the effect of functional trait diversity on multifunctionality has been mainly tested on only a few traits (e.g., two traits in Finney et al., 2017, two traits in Gross et al., 2017, three traits in Lundin et al., 2019) whereas in many cases, when more traits were considered, traits were aggregated to an index implying the loss of ecological information (e.g., Huang et al., 2019; Le Bagousse‐Pinguet et al., 2019; Mensah et al., 2020; Mouillot et al., 2011).

Environmental characteristics influence many aspects of ecology and thus multiple ecosystem functions. For example, for marine sediments, grain size and shell hash content are important drivers of multiple functions in intertidal sandflats, including nutrient regeneration, denitrification, sediment creation, and sediment stability (Hillman et al., 2020; Jones et al., 2011; Thrush et al., 2004, 2013). Therefore, broadening the scope of multifunctionality analysis beyond species metrics to encompass abiotic environmental characteristics can further enhance our understanding of the ecological and mechanical underpinnings of the provision of multiple functions.

In light of the escalating rates of biodiversity loss, holistic ecological insights into species‐function relationships are critically important (IPBES, 2019). Moving away from assessing functions in isolation without compromising on the level of detail of the ecology underpinning multifunctionality is an urgent need in biodiversity–ecosystem function research (Felipe‐Lucia et al., 2020; Snelgrove et al., 2014). Such a task can be performed with network analysis enabling a holistic and transparent way of analyzing the complexity of multifunctional relationships while retaining the insight into the connectivity, strength of individual relationships, pairwise interactions, and recognition of patterns, clusters, and cooccurrences (Morueta‐Holme et al., 2020; Newman, 2010; Segar et al., 2020). The key network components are nodes (variables) and links (that connect the variables, and therefore, establish a relationship). Species or trait groups, environmental characteristics, and functions can be represented as nodes in the network, whereas the links determine relationships between nodes (Bohan et al., 2016; Dee et al., 2017). A recent study by Felipe‐Lucia et al. (2020) demonstrates how networks can be successfully used to assess changes in multifunctionality subject to land use intensity based on multiple ecosystem components (16 trophic groups, 10 ecosystem functions, and 15 ecosystem services).

Studying multifunctionality in heterogeneous and diverse systems while preserving details of individual relationships is a major gap in multifunctionality research, and however, it is important to expand our understanding of multifunctionality mechanics in real‐world systems. To date, most of the multifunctionality methods that do not rely on a single index are best suited to study simple systems with low levels of habitat heterogeneity and low species diversity. For example, the multivariate diversity interactions framework proposed by Dooley et al. (2015) enables insightful analysis into multivariate relationships between species and functions, and however, it was tested on a community of only four species and only three functions. Implementing a multivariate diversity interactions framework on more complex datasets remains challenging (Dooley et al., 2015).

**FIGURE 1:**
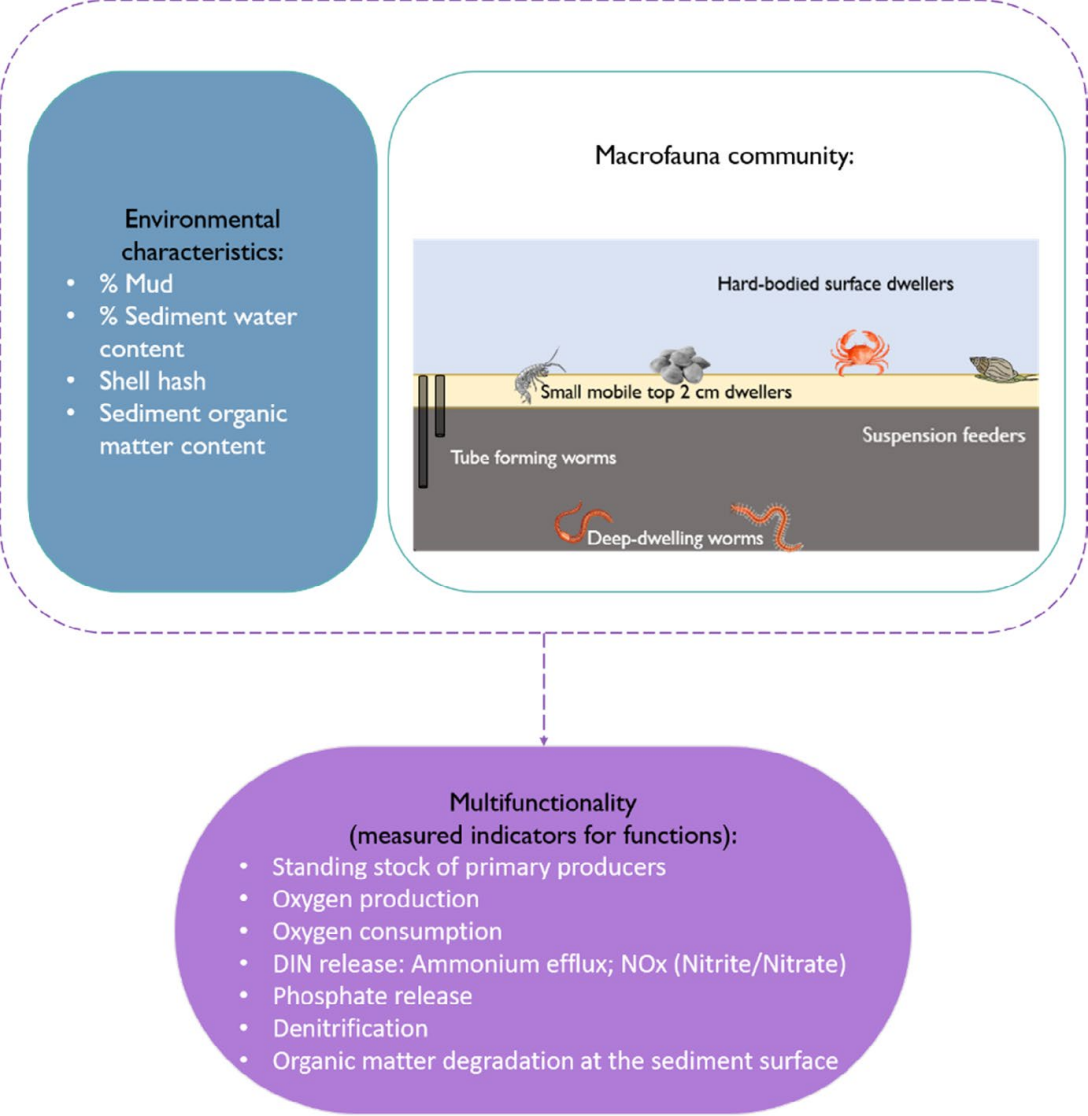
Summary of data used in the multivariate network analysis. The macrofauna community diagram colors represent different sediment layers: yellow—the oxic (~0.5 cm), gray—anoxic sediment layer (>0.5 cm)

In this study, we develop a multivariate network analysis to investigate ecosystem multifunctionality in a diverse intertidal sandflat, Whangateau Harbour, New Zealand (36°18.72′S, 174°46.42′E). We investigated the relationships between eight ecosystem functions, five functional trait clusters defined with 36 macrofaunal species and four key environmental characteristics (Figure 1). Our multivariate network analysis consists of the following key steps: (a) reducing trait dimensionality by identifying trait clusters of functionally similar species and (b) network analysis based on defining the complex relationships between multiple functions and trait clusters, as well as between multiple functions and environmental characteristics. This approach embraces ecological complexity in the multifunctional assessments and is fundamental to our ability to predict the effects of the biodiversity crisis on delivering multiple functions and services.

## 2 MATERIAL AND METHODS

### 2.1 Study location and set‐up

Samples were collected in February/March 2018 on the intertidal sandflats of Whangateau Harbour, New Zealand. Whangateau Harbour covers about 7.5 km^2^ of which, approx. 85% is intertidal soft‐sediment habitat. It is located on the east coast of the North Island (36°20′S 174°45′E). Due to high tidal flushing, small freshwater input and low human population density in the harbor catchment, it is one of the highest quality estuaries within the Auckland region (Cole et al., 2009).

Seven mid‐intertidal sandflat sites (Supplementary materials S1) were selected in the harbor to include differences within macrobenthic community composition (based on the dominant species) and environmental characteristics. At each site, seven experimental plots (replicates) were located approx. 12 m apart. This allowed us to encompass spatial variation within sites and minimize disturbance from sampling. The indicators of ecosystem functions included in the analysis were chosen based on their relevance to describing sandflat's biogeochemistry and its key regulatory functions (Snelgrove et al., 2014; Thrush et al., 2017). Illustration showing the study set‐up and sampling can be found in Supplementary material S1.

#### 2.1.1 Organic matter degradation at the sediment surface

Organic matter degradation within the top 15 cm of sediment was measured using the rapid organic matter assay (ROMA) (O'Meara et al., 2018). The method involves deploying acrylic plates (18 cm × 9 cm × 1.5 cm) in the sediment for a period of 11 days. Each plate has 18 wells (10 mm diam, 9 mm deep) filled with a jelly solution made of 0.029 gC/ml mixture of food‐grade agar, microcrystalline cellulose (CAS 9004‐34‐6; Thermo Fisher), and powdered bran flakes (Edmonds brand). One plate was deployed in each plot (seven plates per study site). Sampling of other variables (see below) took place 6 weeks after collection of ROMA plates to allow the disturbed sediment to settle.

#### 2.1.2 Sediment‐water fluxes: incubation chambers

Benthic incubation chambers were used to measure the net effect of biogeochemical processes that regulate the solute fluxes: dissolved oxygen (DO), and dissolved inorganic nitrogen (DIN: NH_4_^+^, NO_3_^−^, NO_2_) and phosphate (PO_4_^3−^) as well as N_2_ gas (Jones et al., 2011; Thrush et al., 2017). Incubation chambers (17 cm diam, 0.023 m^2^ area) were deployed in the early afternoon and sealed approx. 1 L of ambient seawater above the sediment surface by sinking the chamber edges approx. 2 cm into the sediment. The chambers were sealed so that no air bubbles formed inside the chambers. Each plot contained one light and one dark chamber to allow us to measure ecological processes in the presence and absence of light. Incubations lasted approx. 4 hr around high tide. In addition, at each site, one light one dark 1 L plastic bottles filled with seawater at the same time as the chambers. These water samples were deployed adjacent to the chambers to measure changes in water column DO and nutrient concentrations in the absence of benthic activities. Water samples were extracted from the chambers at the start and end of the incubation period with a syringe (60 ml) via sampling ports. The syringes dedicated to measuring N_2_ gas concentration were gas tight. DO concentrations were measured immediately with a PyroScience optical oxygen meter (FireStingO2 FSO2‐4). Changes in DO were used as a measure of net oxygen production (light chambers) or consumption (dark chambers). DIN and phosphate samples (NH_4_^+^, NO_3_^−^, NO_2_^−^, PO_4_^3−^) were filtered through a Whatman GF/C glass fiber filter (0.8 µm) into 50 ml polyethylene centrifuge tubes and stored on ice in the dark and then frozen at −20℃ on the same day until the laboratory analysis. N_2_ samples were transferred to 12 ml glass vials with added zinc chloride and stored in the dark below ambient temperature in the field and then in the fridge on return to the laboratory.

#### 2.1.3 Sediment sampling

After chamber incubations were complete, benthic chlorophyll *a* concentration, sediment water content, organic matter content, and sediment grain size samples were collected per plot with a syringe core (2.6 cm diam × 2 cm deep cores). All samples were stored on ice and frozen at −20℃ on return to the laboratory the same day. Chlorophyll *a* samples were kept in the dark at all times.

#### 2.1.4 Macrofauna sampling

After chamber incubations were complete, macrofauna and shell hash were sampled for each chamber to measure the activity of the macrofaunal species that directly affected measured functions (two samples per plot, 98 samples in total) using a corer (13 cm diam, 15 cm deep). The bivalves *Austrovenus stutchburyi* and *Paphies australis* were counted and measured on‐site and returned alive due to local restrictions on harvesting. Samples were sieved (500 µm mesh) on‐site and preserved in 70% isopropyl alcohol and stained with rose bengal.

### 2.2 Laboratory analysis

#### 2.2.1 Indicators of ecosystem functions

Sediment samples for chlorophyll *a* were freeze‐dried, and then, 5 g of sediment was extracted in 90% acetone and chlorophyll *a* measured using a Turner Designs 10 AU fluorimeter (Arar & Collins, 1997).

ROMA plates were analyzed within 48 hr from collection, and the organic matter degradation rate at the sediment surface (*C*_o_) was calculated following O'Meara et al. (2018).

DIN and phosphate were measured using flow injection analysis (FIA) with a Lachat Quick‐Chem 8000 automated ion analyzer (Zellweger Analytics, Milwaukee, WI, USA).

Denitrification was determined by analyzing N_2_ concentrations using membrane‐inlet mass spectrometry (MIMS) with a Pfeiffer Vacuum QMS 200 quadrupole mass spectrometer (Kana et al., 1994).

#### 2.2.2 Macrofauna

Macrofauna samples were sorted and identified to the lowest practical taxonomic level (Supplementary materials S2). Macrofaunal species were characterized by their functional traits using fuzzy coding, a procedure that uses proportional scores to show the affinity of a species for different trait modalities (Chevene et al., 1994). 30 trait modalities recognized as important in regulating measured functions were considered and related to the following trait categories: the direction of sediment particle movement, feeding mode, location in or on sediment, sediment structure, mobility, body size, and body hardness (Siwicka & Thrush, 2020; Thrush et al., 2017). For the dominant species, the measurements were recorded for body length, shell length, or body width. The individual size measurements of the dominant species specimens allowed us to represent their functional role more accurately. The other species were size‐classified based on the typical size of an adult (see Supplementary material S2 for more details).

#### 2.2.3 Environmental characteristics

Sediment water content (%) was determined by weight loss of dried sediment (60℃ for 7 days). Sediment organic matter content (%SOM) was determined by weight loss on ignition (450℃ for 4.5 hr). After sediment digestion in 10% hydrogen peroxide and dispersion in 6% Calgon, grain size was determined by filtering wet sediment through the series of sieves of different size (63, 125, 250, 500, and 1,000 μm). The filtered sediment from each sieve was then dried and weighed (Day, 1965). The dry weight of fine particles (>63 μm) determined %mud used in the analysis. Percent shell hash was determined from the dry weight of particles >4 mm from sorted macrofauna samples.

## 3 STATISTICAL ANALYSIS

The data analyses involved multivariate analysis (DistLM) performed in PRIMER‐ e (Clarke & Gorley, 2015); and network analysis of traits (Siwicka et al., 2019) based on species‐trait cooccurrence (Griffith et al., 2016) and environmental network analysis (Bastian et al., 2009). Table 1 outlines the summary of the methodological steps including input data, software requirements, and statistical analysis.

**TABLE 1:** The summary of statistical analysis. Key steps and requirements

Stage	Details of the analysis	Data	Type of analysis	Software	Reference
Reducing trait dimensionality	To reduce trait dimensionality and identify trait clusters composed of functionally similar species (i.e., species that share combinations of trait modalities)	Macrofauna data with assigned functional traits	Network analysis of traits (modularity analysis)	Network analysis of traits consisting of: – cooccur R package, – Gephi	Siwicka et al. (2019) Bastian et al. (2009) Griffith et al. (2016)
Multivariate network analysis	To demonstrate relationships between multiple ecosystem functions, multiple trait clusters, and multiple environmental characteristics	Trait clusters Environmental characteristics Functions	DistLM—to identify the combination of trait clusters and environmental characteristics that best explain individual functions Network analysis— based on the results from DistLM	PRIMER‐ e Gephi	Clarke and Gorley (2015) Griffith et al. (2016)

### 3.1 Reducing trait dimensionality

Macrofauna data and its trait classification were used in this stage of analysis. Trait clusters were identified based on the species‐trait cooccurrence using the network analysis of traits (Siwicka et al., 2019). Network analysis of traits is a two‐stage method that allows for recognizing clusters of species based on a pairwise analysis of species and their traits. First, pairwise associations of species sharing traits were determined using the *cooccur* R package and the strength of species‐trait cooccurrence was determined using Jaccard's coefficient for every pair (see Siwicka et al., 2019 for method details). The second stage involved the environmental network analysis, where all pairwise species associations and their strength were assembled as a network using Gephi software (Bastian et al., 2009; Griffith et al., 2016). Modularity analysis, that is, the analysis of the network partitioning based on the density of links (Blondel et al., 2008), allowed the detection of six clusters that contained functionally similar species (see trait clusters in Figure 1). More details about the cluster composition can be found in Supplementary materials S2.

### 3.2 Multivariate Network Analysis—identifying relationships between multiple functions, trait clusters, and environmental characteristics

DistLM was used to identify the combination of trait clusters and environmental characteristics that best explain individual functions. Nonlinearities in explanatory variables were incorporated by including transformed variables (log_10_(*x* + 1) and square root) and raw variables in the initial model. The selection of both the raw and the squared variable approximates a two‐degree polynomial response. Log‐variables were only allowed to be selected if neither the raw or square transformed variables were selected. First, DistLM was performed to measure the effect of trait clusters in explaining variability in functions using forward selection procedure and adjusted *R* ^2^ stopping criterion. Next, the trait clusters that had proved significant in the first DistLM were used in another DistLM that included the significant trait clusters as a starting position and then added environmental characteristics. Again, DistLM analysis used adjusted *R* ^2^ stopping criterion and forward selection procedure.

The network was assembled in Gephi 0.9.2 (Bastian et al., 2009) based on the DistLM results. The purpose of the network was to identify how biodiversity (traits and combinations of traits) and environment drive patterns in multifunctionality. Thus, links were established between trait clusters and functions; and between environmental characteristics and functions (and not between trait clusters and environment, or functions and environment). The size of the nodes is determined by the total number of links leading out of the node (trait clusters and environmental characteristics) and leading to the node (functions). The dataset was imported as an “edges table” where the “target” (functions) and “source” (trait clusters and environmental characteristics) nodes, as well as the number of outgoing and ingoing connections were determined. Each relationship was directed to show that trait clusters and environmental characteristics influence functions. Each relationship was attributed with a weight that was determined by the % variance explained from DistLM.

## 4 RESULTS

### 4.1 Reducing trait dimensionality

**FIGURE 2:**
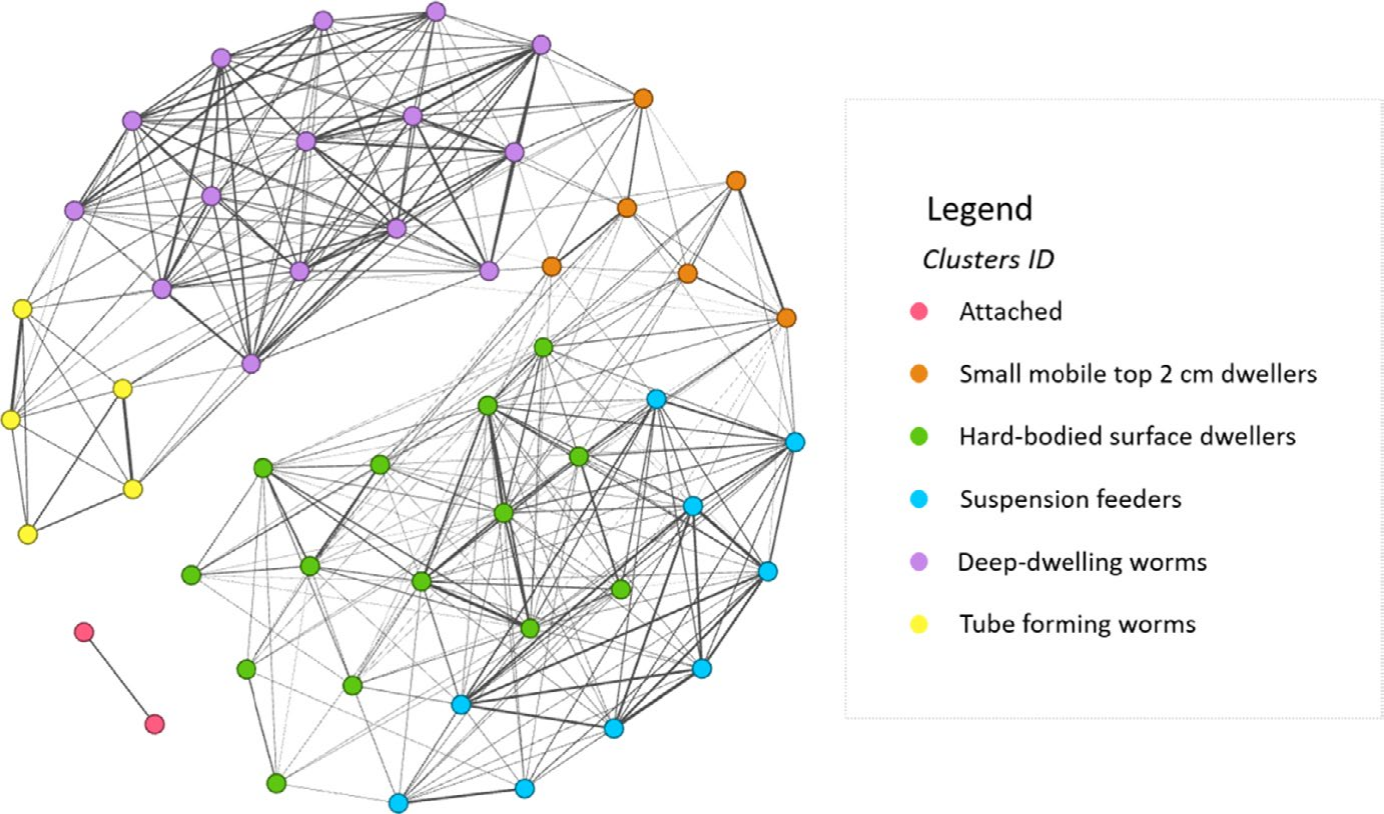
Network analysis of traits (NAT). NAT shows 342 associations between 36 species (nodes). The dominant species are represented as multiple nodes based on the measurement of body length, shell length, or body width. The associations between species are depicted with lines whose thickness indicates the weight based on the number of traits shared between species pairs (Jaccard's coefficient). Six trait clusters (see the legend) were detected from the modularity analysis

Species‐trait cooccurrence analysis identified 342 significant pairwise associations between the 36 species found across the seven study sites. For the dominant species where the measurement was recorded for body length, shell length, and body width (Supplementary materials S2), these species are represented as multiple nodes (e.g., *Austrovenus stutchburyi* is represented by four nodes that represent different shell lengths). The strength of associations (Jaccard's coefficient) ranged from 0.4 for weakly to 1 for strong similarity in the trait space species. For example, *Scoloplos cylindrifer* and *Scolecolepides benhami* species pair has JC strength of 0.84 as these species shared many traits, whereas *Colurostylis lemurum* and *Prionospio aucklandica* species pair has JC strength of 0.4 as they did not share many traits. All 342 pairs and similarity strengths were used in the network analysis and identification of similar clusters. The network analysis of traits visualized the connections and modularity analysis identified six clusters of species that are functionally similar (i.e., they shared traits) (Figure 2). Clusters consisted of species of the dominant traits: (a) attached, (b) small mobile top 2 cm dwellers, (c) hard‐bodied surface dwellers, (d) suspension feeders, (e) deep‐dwelling worms; and (f) tube‐forming worms (see Supplementary materials S2 specific details on clusters composition). The “attached” trait cluster consisted of only one species, an anemone that lives on cockle shells. This trait cluster was disconnected from the rest of the community because this species did not share links (see Figure 2). Given this result and the likely small role of the species in the measured ecosystem function variables, we excluded it from the rest of the analysis.

### 4.2 Multivariate network analysis

**FIGURE 3:**
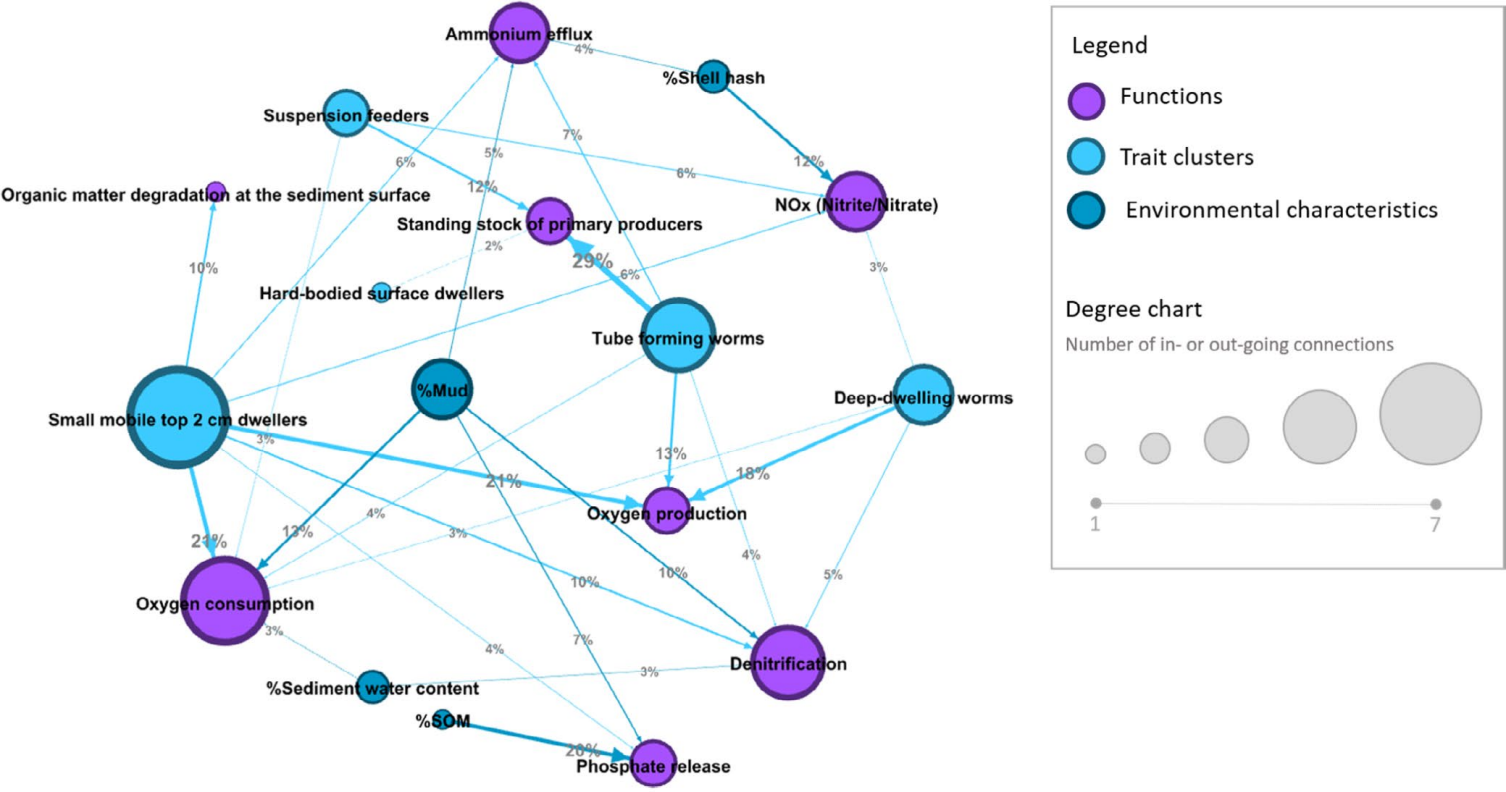
Multivariate network analysis depicting the relationships between multiple functions (purple), trait clusters (blue), and environmental characteristics (teal). Connections in the networks are the individual relationships between multiple functions, trait clusters, and environmental characteristics determined through DistLM (Table 2). The node size indicates the number of in‐ or outgoing connections

Multivariate network analysis showed that trait clusters and environmental characteristics contribute to multiple ecosystem functions creating a network of multiple connections (Figure 3). The connections' strength is based on the % variance explained (DistLM) used to build the network (Table 2). The % variance explained was highest for variables associated with oxygen exchange (oxygen production, 52%; oxygen consumption, 44%) followed by standing stock of primary producers (43%) and net nutrient release from the sediment (phosphate, 32%; denitrification, 32%; NOx, 29%; and ammonium, 22%). The % variance explained was lowest for organic matter degradation at the sediment surface (10%) which was only explained by the abundance in the small mobile top 2 cm dwellers cluster. In general, trait clusters (Table 2A) explained multiple functions better than environmental characteristics (Table 2B).

**TABLE 2:** DistLM showing individual relationships between functions, trait clusters, and environmental characteristics used to populate the network analysis

Trait clusters ‐ Environmental characteristics	Functions							
	Ammonium efflux	NOx (Nitrite/Nitrate)	Phosphate release	Denitrification	Oxygen consumption	Oxygen production	Organic matter degradation at the sediment surface	Standing stock of primary producers
Small mobile top 2 cm dwellers	0.06^b^	0.06^b^	0.04^b^	0.1^a^	0.21^c^	0.21^b^	0.1^b^	–
Suspension feeders	–	0.06^a^	–	–	0.03^a^	–	–	0.12^b^
Deep‐dwelling worms	–	0.03^b^	–	0.05^b^	0.03^b^	0.18^a^	–	
Hard‐bodied surface dwellers	–	–	–	–	–	–	–	0.02^b^
Tube‐forming worms	0.07^b^	–	–	0.04^a^	0.04^b^	0.13^b^	–	0.29^b^
%SOM	–	–	0.2^b^	–	–	–	–	–
%Mud	0.05^a^	–	0.07^a^	0.1^b^	0.13^b^	–	–	–
%Shell hash	0.04^b^	0.14^a^	–	–	–	–	–	–
%Sediment water content	–	–	–	0.03^b^	0.03^b^	–	–	–
(A) *R* ^2^ All significant trait clusters	0.13	0.15	0.04	0.19	0.28	0.52	0.1	0.43
(B) *R* ^2^ All significant environmental characteristics	0.09	0.14	0.27	0.13	0.16	–	–	–
(C) Total (*R* ^2^ (A) & (B) combined)	0.22	0.29	0.31	0.32	0.44	0.52	0.1	0.43

Note

The table shows the proportion of the variability explained by each predictor variable, and the inclusion of variables in the model was determined by forward selection of the adjusted *R* ^2^ selection criterion. For each function relationship, the result was obtained by selecting: (^a^) only untransformed variable; (^b^) only a log transformation, only a square transformation, or the untransformed variable and the square transform variable; (^c^) log transformed variable together with either the untransformed variable or the square transformed variable.

The network analysis visualizes the relationships between multiple functions, trait clusters, and environmental characteristics (Figure 3). The analysis of the distribution of the connections demonstrated that among the trait clusters, small mobile top 2 cm dwellers were the node with the highest number of outgoing connections and contributed to explaining seven out of eight functions. High connectivity of tube‐forming worms (connected to 5 functions), deep‐dwelling worms (connected to 4 functions), and suspension feeders (connected to 3 functions) in defining multiple functions were also observed. Hard‐bodied surface dwellers were only important to the standing stock of primary producers. Among environmental characteristics, %mud has the highest connectivity and contributed to four out of eight functions.

## 5 DISCUSSION

Increasing our understanding of the ecology and the environment underpins the provision of ecosystem functions requires a detailed and holistic analysis of ecosystem multifunctionality (Snelgrove et al., 2014). Our study represents multifunctionality as a network of relationships between multiple functions, trait clusters, and environmental characteristics (Figure 3). Different combinations of trait clusters and environmental characteristics were important in explaining different functions (Figure 3). Most highly explained functions related to nutrients fluxes and oxygen regulation (Table 2). Among trait clusters, the most functionally prevalent were small mobile top 2 cm dwellers then tube‐forming worms, deep‐dwelling worms, and suspension feeders. The most important environmental characteristic was %mud.

### 5.1 Investigating ecosystem multifunctionality with multivariate network analysis

The network analysis revealed how different trait clusters contribute to different ecosystem functions. Importantly, the network showed that most of the trait clusters (i.e., small mobile top 2 cm dwellers, attached species, deep‐dwelling worms, tube‐forming worms and suspension feeders) influenced a wide array of functions. This result confirms that species, through their biological activity, affect multiple ecosystem functions and that a single function is a product of multiple traits (Byrnes et al., 2014; Siwicka et al., 2019). For example, denitrification was influenced by small mobile top 2 cm dwellers, deep‐dwelling worms and tube‐forming worms (19% of variability explained) as well as %mud and %sediment water content (13% of variability explained). The observed importance of the macrofauna community to denitrification supports the findings of O'Meara et al. (2020), who demonstrated that small, abundant tube building worms enhanced denitrification rates. Denitrification is an important function provided by estuarine ecosystems. It maintains ecosystem health by removing harmful excess of nitrogen (Barbier et al., 2011; Thrush et al., 2013). Macrofaunal communities, through their functional traits, such as mobility, feeding mode, sediment particle movement, and creation of sediment structures, affect microbial processes influencing denitrification (Douglas et al., 2016; Vieillard et al., 2020). However, the major stressors, including terrestrial sediment and nutrient run‐off, cause significant changes to oxygen availability leading to eutrophication and mass mortalities (Kennish, 2002; Thrush et al., 2013), which alter both community composition and environmental characteristics of the benthos.

Our results show that %mud was the strongest predictor of functions among environmental characteristics, working to decrease functionality. Increasing sedimentation rates have major impacts on macrofaunal communities and can alter species behavior, change distribution, and densities and even cause localized extinction (Thrush et al., 2004).

The results from multivariate network analysis explained between 10% and 50% of the variability in individual functions. Similar scale of effect was observed in other studies on multifunctionality in benthic systems. For example, Villnäs et al. (2013) using distance‐based linear models demonstrated that the benthic trait composition of macrobenthic fauna alone explained only 9% of variation in ecosystem functioning. Intertidal sandflats are highly dynamic systems, and their functionality is regulated by many biogeochemical and physical processes (Barbier et al., 2011; Kennish, 2002). In our study, we were interested in the effect of macrofauna community, their traits, and key environmental characteristics on ecosystem multifunctionality. However, other variables that influence multifunctionality exist that were beyond the scope of this research. For example, the effect of denitrifying bacteria on denitrification rates that convert reactive nitrogen into N_2_ gas, effectively removing N from the system (Vieillard et al., 2020). Macrofaunal organisms through their biological activity such as structure building and sediment particle movement introduce organic carbon and oxygen to deeper layers of sediment where denitrifiers reside (Siwicka & Thrush, 2020), and therefore, their role, although indirect, is critical in mediating denitrification rates (Vieillard & Thrush, 2021). Low explanatory power of variables should not discourage us from studying the range of drivers that regulate multifunctionality. Importantly, method transparency as offered by the network approach, thorough understanding of the multifunctional relationships and appropriate interpretation of the results is essential in multifunctionality studies in complex systems.

The multivariate network analysis also provides insights into functional redundancy, that is, the community's ability to maintain functionality under changing conditions (Walker, 1992). By assessing the density of connections between species that formed trait clusters (Figure 2) and clusters that underpin functions (Figure 3), we can determine weakly connected components that are potentially more sensitive to environmental stressors. For example, Siwicka et al. (2019) show that experimentally increased nitrogen levels induced changes in the weakly connected clusters of the community network indicating that the community started to homogenize with increasing nitrogen loading. Changes in the macrofaunal community from external stressors (sediment and nutrients deposition) can forewarn changes in functionality and a loss in multiple ecosystem services. The awareness of redundancy patterns and macrofaunal species' role in the performance of the individual functions can help prioritize the protection or restoration of the functionally important species in the management strategies to maintain overall ecosystem functionality.

### 5.2 Network analysis in complex systems—beyond a single multifunctionality index

Although functional traits are generally good predictors of functions (Cadotte et al., 2011; Petchey et al., 2004; Reiss et al., 2009), they remain underrepresented in multifunctionality assessments (Gross et al., 2017; Le Bagousse‐Pinguet et al., 2019; Mensah et al., 2020). Even when a trait‐based approach has been utilized, the number of traits considered has been limited (e.g., two or three traits (see Finney et al., 2017; Gross et al., 2017; Lundin et al., 2019) or traits are represented as an index (e.g., *F*_Ric_, *F*_Eve_, *F*_div_ (see Mouillot et al., 2011; Huang et al., 2019; Le Bagousse‐Pinguet et al., 2019; Mensah et al., 2020)). Network analysis is a visual tool that enables studying ecosystems holistically, and the relationships between the nodes are clear and transparent, even when dealing with complex and diverse ecosystems such as the intertidal sandflat presented in our study. The network analysis allowed us to detect patterns and cooccurrences using network analysis of traits approach (Siwicka et al., 2019) and to gain insight into the relative importance of individual connections between functions, trait clusters, and environmental characteristics (multivariate network analysis). Our framework was tested with a high level of functional diversity consisting of 30 trait modalities (Supplementary materials S2). Using the network analysis of traits, we established the within‐trait species similarities and identified trait clusters (named based on their dominant traits) (Figure 2). Network analysis of traits reduced the level of trait dimensionality, but it preserved the ecological information underpinning functions and it included all functionally significant traits in the next stages of the analysis, making the final results more informative.

Our approach provides both an exploratory framework to investigate relationships and the potential to test hypothesis about specific changes in network architecture that arise in the analysis of trait clusters, environmental factors, and multiple ecosystem functions. Our multivariate network analysis aims to explore the concept of multifunctionality and “tell the story” by delivering insights into the ecological underpinnings of multiple functions. This complements other approaches such as structural equation modelling (*SEM*) that have stricter data requirements (Grace et al., 2010). The observations from our framework inform about the complex nature of connections, patterns, and cooccurrences within the system and can lead to more specific hypothesis that can be tested in the future research.

Multivariate network analysis, unlike standard species richness‐based multifunctionality methods that aggregate measures of ecosystem functions (Bradford et al., 2014), allows us to trace and investigate each relationship to the level of individual species, traits, or functional groups. Such transparency helps to reduce the risk of the phenomenon called “the black box” that is often used as a metaphor in the context of ecological research where the internal workings of the systems are not clear and cannot be readily understood (Herman et al., 2019). The transparency of connections as presented in our multivariate network analysis helps explaining the complexity behind ecosystem multifunctionality and can promote the use of ecosystem multifunctionality framework among decision‐makers.

### 5.3 A need for expanding the range of methods for studying ecosystem multifunctionality

Our framework offers a new way of holistic and transparent viewing of multifunctionality and goes beyond a simple index. Aggregating multiple functions into an index is a common practice, with 84% of studies to date presenting multifunctionality as a single metric (Hölting et al., 2019). As with any comparison between a multivariate versus univariate approach, there are always trade‐offs in specificity, generality and interpretability. While the messages revealed from multivariate network analysis are difficult to compare with “classical” index‐based multifunctionality studies, ultimately, the focus and strength of our framework provide more insight into ecological relationships within studied ecosystems. As demonstrated in our study, the network analysis emphasizes that an act of understanding multifunctionality in real‐world ecosystems is an art of balancing our pursuit of simplicity while recognizing and embracing inherent ecosystem complexity.

## CONFLICT OF INTEREST

There are no conflicts of interest.

## AUTHOR CONTRIBUTIONS

**Ewa Siwicka:** Conceptualization (lead); data curation (lead); formal analysis (lead); investigation (lead); methodology (lead); software (lead); visualization (lead); writing‐original draft (lead); writing‐review & editing (equal). **Simon F. Thrush:** Conceptualization (supporting); data curation (supporting); formal analysis (supporting); funding acquisition (lead); investigation (equal); methodology (supporting); project administration (equal); resources (equal); software (supporting); supervision (lead); validation (equal); visualization (supporting); writing‐original draft (supporting); writing‐review & editing (supporting). **Rebecca Gladstone‐Gallagher:** Formal analysis (supporting); supervision (supporting); validation (supporting); writing‐original draft (supporting); writing‐review & editing (supporting). **Judi E. Hewitt:** Formal analysis (supporting); methodology (supporting); software (supporting); supervision (supporting); validation (supporting); writing‐original draft (supporting); writing‐review & editing (supporting).

## ETHICAL APPROVAL

Hereby, I Ewa Siwicka consciously assure that for the manuscript “Beyond the single index: Investigating ecological mechanisms underpinning ecosystem multifunctionality with network analysis” the following is fulfilled: (1) This material is the authors' own original work, which has not been previously published elsewhere; (2) the paper is not currently being considered for publication elsewhere; (3) the paper reflects the authors' own research and analysis in a truthful and complete manner; (4) the paper properly credits the meaningful contributions of coauthors and coresearchers; (5) the results are appropriately placed in the context of prior and existing research; and (6) all sources used are properly disclosed (correct citation). Literally copying of text must be indicated as such by using quotation marks and giving proper reference; (7) all authors have been personally and actively involved in substantial work leading to the paper and will take public responsibility for its content.

## Supporting information

Supplementary Material

Supplementary Material

## Data Availability

Data available from the Figshare Digital Repository: https://doi.org/10.17608/k6.auckland.13151066. URL: https://figshare.com/articles/dataset/Multifunctional_Network_Analysis/13151066.
