# Supplementary material for: Beyond the single index: Investigating ecological mechanisms underpinning ecosystem multifunctionality with network analysis
Source: Ecol Evol. 2021 Aug 24;11(18):12401–12. doi: 10.1002/ece3.7987 (PMC8462174; doi:10.1002/ece3.7987)
Supplement: Supplementary file 1 — Supplementary Material [file ECE3-11-12401-s002.docx]

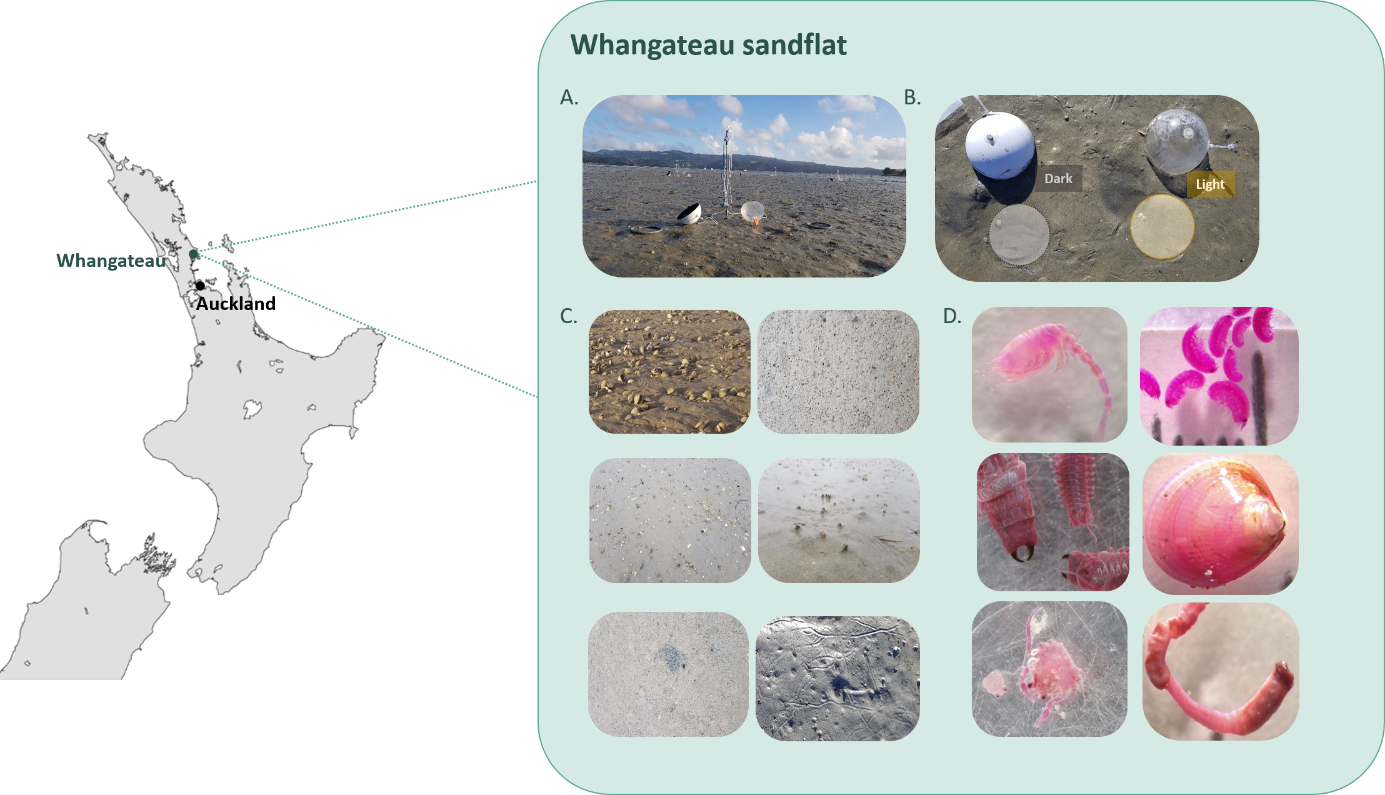


Figure S3.1. Location of the Whangateau Harbour, New Zealand. A – shows a set-up of incubation chambers in a single plot; B – each plot consisted of two chambers (light and dark); C – heterogeneity between sites in sediment features and dominant species; D – examples of macrofaunal species found in the sediment across sites.


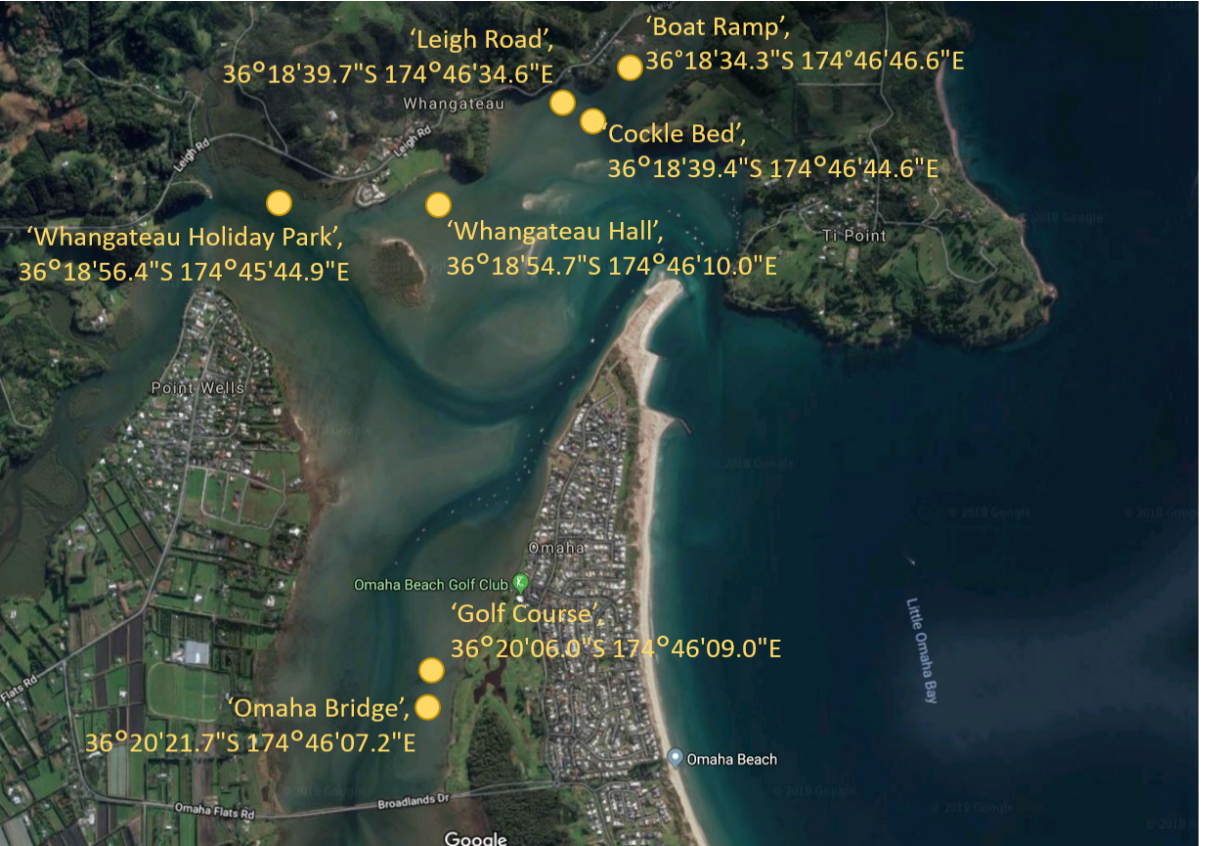


Figure S3.2. Individual sites location.


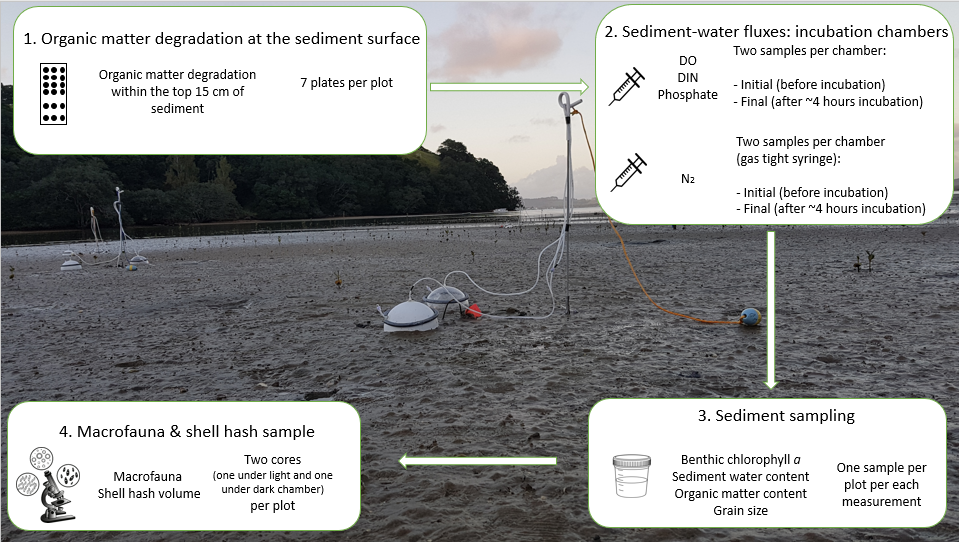


Figure S3.3. Study set-up: collected samples.
