# Supplementary material for: Beyond the single index: Investigating ecological mechanisms underpinning ecosystem multifunctionality with network analysis
Source: Ecol Evol. 2021 Aug 24;11(18):12401–12. doi: 10.1002/ece3.7987 (PMC8462174; doi:10.1002/ece3.7987)
Supplement: Supplementary file 2 — Supplementary Material [file ECE3-11-12401-s001.docx]

Table S2. Data summary used in the multivariate network analysis

| **Species** | | **Traits** | | **Trait clusters of species sharing combinations of traits (determined by network analysis of traits)** | | **Environmental characteristics** | **Ecosystem functions** | |
| --- | --- | --- | --- | --- | --- | --- | --- | --- |
| *Typical adult size* | *Size of dominant species based on the body width, body length or shell length:*  *^1^ <1/>1 mm*  *^2^ <3/ >3 mm*  *^3^ <5/ >5 mm*  *^4^ <10/>10 mm*  *^5^ <1/1-9/10-19/20-29/>30 mm* | *Trait category* | *Trait modality* | *Cluster ID*  *(Based on dominating traits of species)* | *Species ID*  *(see 1^st^ column of this table)* | *(Determined from the sediment samples)* | *Function* | *Indicator:*  ** Light chamber*  *** Dark chamber*  *^ Sediment* |
| 1. Amphipoda 2. Mysidacea 3. *Colurostylis lemurum* 4. Ostracoda 5. *Austrohelice crassa* 6. *Halicarcinus whitei* 7. *Diloma subrostrata* 8. *Notoacmea scapha* 9. *Chiton glaucus* 10. *Aricidea* sp. 11. *Paradoneis lyra* 12. *Phoronis* sp. 13. *Heteromastus filiformis* 14. *Asychis* sp. 15. *Capitella* sp. 16. Maldanidae 17. *Boccardia syrtis* 18. *Minuspio* sp. 19. Exogoninae 20. *Scoloplos cylindrifer* 21. *Scolecolepides benhami* 22. Oligochaeta 23. Lumbrineridae 24. Nemertea | 1. *Anthopleura auradiata*^3^ 2. Isopoda*^2^* 3. *Austrovenus stutchburyi^5^* 4. *Paphies australis^4^* 5. *Linucula hartvigiana^2^* 6. *Macomona liliana^4^* 7. *Zeacumantus lutulentus^3^* 8. *Zeacumantus subcarinatus^3^* 9. *Cominella glandiformis^3^* 10. *Prionospio aucklandica ^1^* 11. *Platynereis australis^1^* 12. *Orbinia paplliosa^1^* | Direction of sediment particle movement | - Surface to depth - Depth to surface - Surface to surface - Depth to depth | Attached | 25 | % Sediment organic matter (SOM) | Standing stock of primary producers | Chl *a*^^^ |
|  |  | Feeding mode | - Suspension - Deposit - Predator - Scavenger - Grazer | Small mobile top 2 cm dwellers | 1, 2,26, 3, 19 | % Mud | Oxygen production | DO* |
|  |  | Location in or on sediment | - Surface - Attached - Top 2 cm - Deep | Hard-bodied surface dwellers | 4, 5, 6, 29, 31, 32, 33,7, 8, 9 | % Shell hash | Oxygen consumption | DO** |
|  |  | Sediment structure | - Permanent burrow - Simple hole or pit - Tube structure - Mound - Trough - Trampling | Suspension feeders | 27, 28, 30 | % Sediment water content | DIN release:   - Ammonium efflux - NOx (Nitrite/Nitrate) | NH_4_+**  (NO_2_^−^ /NO_3_^−^)** |
|  |  | Mobility | - Sedimentary or limited mobility - Motile | Deep-dwelling worms | 10, 11, 34, 13, 15, 35, 36, 20, 21, 22, 23, 24 |  | Phosphate release | PO_4_^3-^** |
|  |  | Body size | - Small - Medium - Large | Tube forming worms | 12, 14, 16, 17, 18 |  | Denitrification | N_2_** |
|  |  | Body hardness | - Soft-bodied - Rigid - Calcified |  |  |  | Organic matter degradation at the sediment surface | C_0_^ |
